# Multiple Effects of Berberine Derivatives on Colon Cancer Cells

**DOI:** 10.1155/2014/924585

**Published:** 2014-06-18

**Authors:** Luis Miguel Guamán Ortiz, Micol Tillhon, Michael Parks, Ilaria Dutto, Ennio Prosperi, Monica Savio, Andrea G. Arcamone, Franco Buzzetti, Paolo Lombardi, Anna Ivana Scovassi

**Affiliations:** ^1^Istituto di Genetica Molecolare CNR, 27100 Pavia, Italy; ^2^Departamento de Ciencias de la Salud, Universidad Técnica Particular de Loja, San Cayetano Alto, Calle París, 1101608 Loja, Ecuador; ^3^Department of Molecular Medicine, Immunology and General Pathology Unit, University of Pavia, Via Ferrata 9, 27100 Pavia, Italy; ^4^Naxospharma srl, Via Giuseppe Di Vittorio 70, 20026 Novate Milanese, Italy

## Abstract

The pharmacological use of the plant alkaloid berberine is based on its antibacterial and anti-inflammatory properties; recently, anticancer activity has been attributed to this compound. To exploit this interesting feature, we synthesized three berberine derivatives, namely, NAX012, NAX014, and NAX018, and we tested their effects on two human colon carcinoma cell lines, that is, HCT116 and SW613-B3, which are characterized by wt and mutated *p53*, respectively. We observed that cell proliferation is more affected by cell treatment with the derivatives than with the lead compound; moreover, the derivatives proved to induce cell cycle arrest and cell death through apoptosis, thus suggesting that they could be promising anticancer drugs. Finally, we detected typical signs of autophagy in cells treated with berberine derivatives.

## 1. Introduction

Berberine (BBR) is an isoquinoline quaternary alkaloid present in many medicinal plants such as* Hydrastis canadensis*,* Berberis aristata*,* Coptis chinensis*,* C. rhizome*,* C. japonica*,* Phellodendron amurense*,* P. chinense *Schneid., and other plant species used around the world in traditional medicine. Plants containing BBR have been used for the prevention and treatment of many diseases, including gastrointestinal infections, abdominal pain and diarrhea, hyperglycemia, hyperlipidemia, metabolic syndrome, polycystic ovary syndrome, obesity, fatty liver, and coronary artery disorders [1–4].

**Figure 1:**
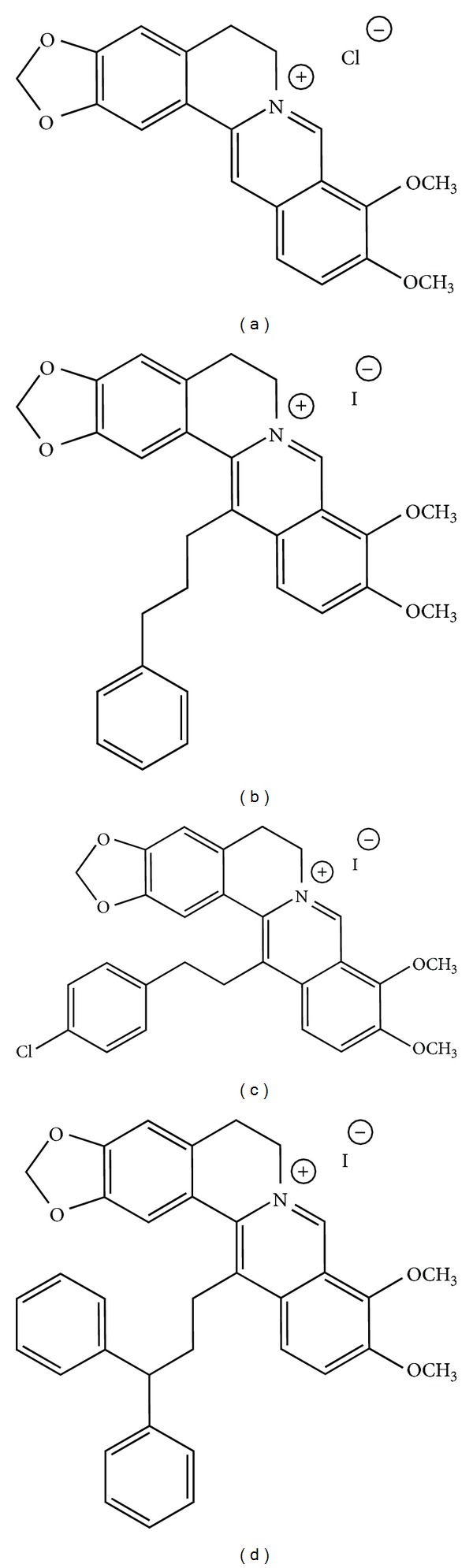
Molecular structure of berberine (a), NAX012 (b), NAX014 (c), and NAX018 (d).

It is well known that some alkaloids, such as the topoisomerase I inhibitors camptothecin and vinblastine (both isolated from plants), which interact with tubulin, have already been successfully used as chemotherapeutic drugs. Accordingly, also BBR proved to have anticancer effects [3–12] on different tumor cell lines. The nitrogen atom present at the 7-position of the alkaloid skeleton of the BBR molecule (Figure 1(a)) has a positive charge possibly responsible for its ability to form strong complexes with either DNA or RNA [6, 13, 14], thus inducing DNA damage and promoting telomerase inhibition and topoisomerase poisoning [15, 16]. Moreover, BBR can suppress gene transcription by affecting the association between the TATA-binding protein and the TATA box in the gene promoters [17], and regulating the expression of Bcl-2-family members, such as Bax, Bcl-2, and Bcl-xL, which play crucial roles in apoptosis [18, 19]. Additionally, the general antioxidant and anti-inflammatory properties of BBR has been correlated to the inhibition of cyclooxygenase-2 (COX-2) [20, 21]. These events may lead to cell cycle arrest, induce cell death* via* apoptosis, and also activate autophagy [22].

The structure of BBR represents a biologically interesting skeleton and also an attractive natural lead compound for the introduction of various chemical modifications in appropriate positions, in search for more selective, discriminated, and narrowed medical applications [13]. Therefore, aiming at ameliorating the anticancer properties of BBR, we have designed and synthesized BBR derivatives: NAX012, NAX014, and NAX018 (Figures 1(b)–1(d)) which are characterized by the presence of aromatic groups bonded to the 13-position of the parent alkaloid skeleton through a hydrocarbon linker, to possibly create a geometric propensity for additional stacking-type, noncovalent, aromatic interactions (intramolecular and/or molecule-cellular target). Aromatic interactions are ubiquitous in nature, and their geometry is relevant for the molecular interactions within cell components possibly with nucleic acids [23, 24].

To deeper investigate the biological effects of these compounds, we performed several cellular and molecular assays for evaluating cell proliferation, cell cycle distribution, apoptosis, and autophagy in cells treated with the BBR derivatives. The analysis was performed on the colon carcinoma cell lines HCT116 and SW613-B3, which present a different status of the oncosuppressor* p53*, with HCT116 being wild type and SW613-B3 mutated. As we previously reported [25], SW613-B3 cells are characterized by a mutation leading to the CGT to CAT transition at codon 273 of* p53*, resulting in the substitution of the “hot spot” aa His with Arg within DNA binding domain, thus blocking the transcriptional activity of* p*5*3*.

## 2. Materials and Methods

### 2.1. Berberine and Its 13-Arylalkyl Derivatives NAX012, NAX014, and NAX018

The 13-arylalkyl berberine derivatives were designed, synthesized, and characterized by Naxospharma [US Pat. 8,188,109 B2 to Naxospharma srl, granted on May 29, 2012, first published as US 2011/0015222 A1 on January 20, 2011, priority date July 20, 2009], starting from commercial berberine chloride hydrate (*ca.* 17% H_2_O), which was purchased from Shanghai Trust & We, China (Figure 1(a)). The purity (>95%) of the derivatives was assessed by HPLC on a Jasco system LC-2000 series (Jasco, Europe) with an Agilent Eclipse XDB-C18 (4.6 mm × 150 mm × 3.5 mm) column (Agilent Technologies, USA). The flow rate of the mobile phase (50% water, 50% acetonitrile plus 0.1% trifluoroacetic acid) was maintained at 1 mL/min and absorbance was measured at 235, 265, 340, and 420 nm.

*NAX012 (Figure 1(b))*. NMR (200 MHz, DMSO-d_6_) *δ*: 9.88 (s, 1H), 8.19 (d, 1H), 8.20 (d, 1H), 7.70 (m, 1H), 7.29 (s, 1H), 7.16 (s, 1H), 6.18 (s, 2H), 4.80 (m, 2H), 4.00 (s, 3H), 3.10 (t, 2H), 2.50 (m, 4H).

*NAX014 (Figure 1(c))*. NMR (200 MHz, DMSO-d_6_) *δ*: 10.02 (s, 1H), 9.87 (s, 1H), 9.86 (s, 1H), 8.33 (d, 1H), 8.24 (d, 1H), 7.95 (d, 1H), 7.38 (d, 2H), 7.22 (d, 2H), 7.05 (s, 2H), 6.16 (s, 2H), 4.12 (s, 3H), 4.11 (s, 3H), 4.02 (m, 2H), 3.29 (t, 2H), 2.88 (m, 4H).

*NAX018 (Figure 1(d))*. NMR (200 MHz, DMSO-d_6_) *δ*: 9.90 (s, 1H), 8.15 (d, 1H), 8.10 (d, 1H), 7.20 (m, 10H), 7.10 (s, 2H), 6.20 (s, 2H), 4.80 (m, 2H), 4.15 (s, 3H), 4.10 (s, 3H), 4.0 (d, 1H), 3.2 (t, 2H), 2.5 (m, 6H).

### 2.2. Cell Culture and Treatments

Human colon carcinoma HCT116 and SW613-B3 cells and normal fibroblasts FO46 (the origin of which has been previously described [27]) were grown at 37°C and 5% CO_2_ atmosphere, in Dulbecco's modified Eagle's medium (DMEM) for SW613-B3 and FO46 cells or RPMI medium (HCT116 cells), supplemented with 10% FBS, 0.1 mg/mL penicillin, 100 U/mL streptomycin, 2 mM glutamine, and 2% sodium pyruvate (all reagents were from Euroclone, Milano, Italy). Twenty-four hours after seeding, cells were treated for 24 h either with etoposide (Sigma Aldrich, Milano, Italy, stock solution: 50 mM in DMSO) or HMA (5-(*N*,*N*-hexamethylene)amiloride; Sigma Aldrich, stock solution: 80 mM in DMSO), BBR, or BBR derivatives NAX012, 014, and 018 (stock solutions: 10 mM in DMSO), followed by a 24 h recovery in drug free medium. In some experiments, cells were pretreated with 2.5 mM 3-methyladenine (3 MA, Sigma Aldrich, stock solution: 100 mM in DMSO) for 4 h. In general the final concentration of DMSO in culture medium was <0.2% (v/v) and did not affect the tested activities. Under some experimental conditions, a fraction of treated cells tended to detach; this population was analyzed separately or in combination with attached cells, as specified for each assay.

### 2.3. Morphological Analysis

For brightfield microscope observation, cells grown in 3.5 cm diameter Petri dishes (5 × 10^4^/mL) were treated with 10 *µ*M BBR or BBR derivatives for 24 h. At the end of the treatment, cells were observed using an Olympus IX71 microscope equipped with a 10x objective and images were acquired with a digital camera Cool SNAPES (Photo Metrics, CA, USA), using the MetaMorph acquisition software; Adobe Photoshop 9.0.2 was used as elaborating software.

### 2.4. Viability Assays

The effect of drugs on cell proliferation was evaluated by two different procedures, that is, the MTT metabolic viability assay, which measures mitochondrial activity, and the quantification of the amount of DNA released from cells after alkaline lysis, that is, proportional to the cell number [28]. For the MTT assay, cells were seeded in 96-multiwell plates at the density of 10^3^ in 100 *µ*L/well and, 24 h later, treated with 1 *µ*M or 10 *µ*M BBR and BBR derivatives for 24 h followed by a 24 h recovery in drug free medium. In some experiments, cells were preincubated with 2.5 mM 3MA for 4 h. Parallel samples were incubated with 0.1% DMSO to evaluate the possible effect of the solvent. At the end of the incubation, 20 *µ*L of Cell Titer 96 Aqueous One Solution cell proliferation reagent (Promega Italia, Milano, Italy) were added to each well. The plates were then maintained for 4 h at 37°C; the absorbance of each sample was measured with a microplate reader (EZ Read 400, Biochrom, Cambridge, UK) at wavelength of 492 nm. Experiments were performed in quadruplicate and repeated three times. Data obtained from untreated cells were used as reference values (considered as 100%) to normalize the absorbance of treated samples.

For the DNA release assay, cells were seeded in 6 cm diameter Petri dishes at a density of 5 × 10^4^ cells/mL and, 24 h later, treated for 24 h with 1 or 10 *µ*M BBR derivatives, and further incubated for 24 h in drug free medium. In addition, controls and samples incubated with 0.1% DMSO were also processed, according to a published procedure [28]. Three independent experiments were carried out. Statistical analysis was performed and data were presented as mean ± S.D.

### 2.5. Clonogenic Assay

To evaluate colony forming ability, 2.5 × 10^2^ cells/mL were seeded in 6 cm diameter Petri dishes and, 24 h later, treated with BBR derivatives for 24 h and further grown in complete medium for 10 days to allow colony formation by surviving cells [28]. Colonies with more than 50 cells were counted. The number of colonies of treated cells was compared to that of control samples, and clonogenic efficiency was expressed as the percentage with respect to untreated cells. Experiments were performed in duplicate and repeated three times.

### 2.6. Cell Cycle Analysis

To evaluate cell cycle distribution of the whole cell population, cells were seeded in 10 cm diameter Petri dishes (10^6^ cells/dish), grown in complete medium for 24 h, and treated with 10 *µ*M BBR derivatives for 24 h. Samples were processed as described [29] and analyzed using a Coulter Epics XLII flow cytometer (Beckman Coulter, Milano, Italy); for each sample, 10^4^ cells were measured. The fluorescence intensity was converted into histograms, and the percentage of cells in each phase of the cell cycle was calculated with XLII software. Experiments were repeated three times.

### 2.7. Immunofluorescence Experiments

Cells were seeded on coverslips (5 × 10^4^ cells/mL), treated with drugs for 24 h and then fixed with cold paraformaldehyde (2% in PBS) for 20 min, postfixed overnight with 70% ethanol at −20°C, and permeabilized with 0.1% Triton X-100 in PBS. Samples were then incubated with the MAb to mtHSP70 (JG1, Alexis, Vinci Biochem, Vinci, Italy, diluted 1 : 50) according to [28]. For poly(ADP-ribose) analysis, fixation and incubation with the monoclonal antibody 10H (ALX-804-220, Alexis, diluted 1 : 100) and with the appropriate secondary antibody were performed as previously described [26]. For* p53* and* p21* analysis, cells were lysed with hypotonic buffer (10 mM Tris-HCl, 2.5 mM MgCl_2_, 10 mM *β*-glycerophosphate, 0.1% Igepal, 0.2 mM PMSF, and 0.1 mM Na_3_VO_4_) and washed with washing buffer (10 mM Tris-HCl, 2.5 mM MgCl_2_, 10 mM *β*-glycerophosphate, 0.2 mM PMSF, and 0.1 mM Na_3_VO_4_). Then, samples were processed as described; the visualization of* p53* and* p21* proteins has been achieved using the MAb DO7 (Dako, Glostrup, Germany) and the polyclonal N-20 (Santa Cruz), respectively [30]. Three independent experiments were performed.

*In situ* conversion of LC3 form I to form II was visualized by immunofluorescence after fixation of cells with cold paraformaldehyde (4% in PBS) for 15 min in ice and permeabilization with cold acetone for 5 min. After washings with PBS, samples were incubated with bovine serum albumin (4% in PBS) for 10 min and with the polyclonal antibody 2775 to LC3 (Cell Signaling, diluted 1 : 100) for 1 h at 37°C followed by the incubation with the appropriate secondary antibody [26]. As a positive control of autophagy, cells were treated for 24 h with 20 *µ*M HMA [26]. Three independent experiments were performed. Cells were observed using a fluorescence microscope Olympus BX51, equipped with a 60x objective. The images were acquired with a digital camera Camedia C4040 (Olympus); the quantification of autophagic vacuole punctuation has been performed; Adobe Photoshop was used as elaborating software.

### 2.8. Statistical Analysis

The ANOVA and Dunnett's multiple comparison tests have been applied. The statistical analysis was performed using GraphPad Prism 5.0.

### 2.9. Western Blotting

Protein expression in HCT116 and SW613-B3 cells treated for 24 h with BBR derivatives was evaluated by western blotting according to a described protocol [26]. After running and transferring of proteins onto nitrocellulose, membranes were incubated overnight at 4°C, or 3 h at room temperature, with MAbs against the following proteins: PARP-1 (C2-10 Alexis, diluted 1 : 1000); total caspase 3 (31A1067 Alexis, diluted 1 : 250); and *γ*-tubulin (GTU-88 Sigma, diluted 1 : 10,000). A polyclonal antibody against total caspase 8 (BioVision, Milpitas, USA, diluted 1 : 1000) was used. Autophagy was monitored through the marker LC3 using the polyclonal antibody 2775 (Cell Signaling, diluted 1 : 1000 [31]). For* p53* and* p21* analysis, a previously described procedure has been applied, based on the use of the same MAb described in the immunofluorescence section [30]. The appropriate HRP-conjugated (anti-mouse or anti-rabbit) secondary antibody (Jackson Immuno Research, Suffolk, UK, diluted 1 : 10,000) was applied for 45 min at room temperature. All antibodies were diluted in TBS (140 mM NaCl, 100 mM Tris-HCl, pH 7.5) containing 5% skimmed milk and 0.1% Tween-20. Visualization of the immunoreactive bands was achieved using a chemiluminescent substrate (Immun-Star WesternC Chemiluminescent Kit, Bio Rad Laboratories, Segrate, Italy). Three independent experiments were performed.

### 2.10. Internucleosomal DNA Degradation

For DNA ladder visualization, control and treated samples (2.5 × 10^6^ cells) were processed as reported [28]. Cells treated with 100 *µ*M etoposide for 24 h were used as positive DNA ladder occurrence [31]. Pictures were taken with a photographic digital camera Kodak DC290 (Rochester, NY, USA).

## 3. Results and Discussion

Within the frame of an active search for compounds with cytotoxic effect on cancer cells, berberine (BBR) has been described as a promising drug; thus, new 9-O-derivatives [32] and 13-substituted BBR derivatives [33, 34] were designed and synthesized. The present work aimed to evaluate the biological effects of BBR and three derivatives (NAX012, NAX014, and NAX018) characterized by aromatic moieties bonded to the 13-position of BBR through a linker of variable length. The experiments were carried out on human colon cancer cell lines HCT116 and SW613-B3.

### 3.1. Berberine Derivatives Affect Cell Morphology

**Figure 2:**
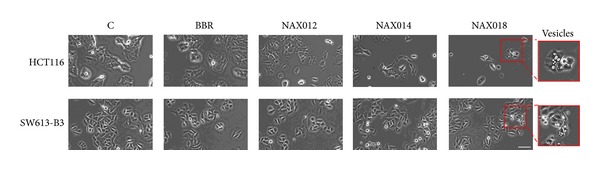
Effect of BBR and its derivatives on cell morphology. Bright field images of untreated (C) HCT116 and SW613-B3 cells and of samples treated with 10 *µ*M BBR, NAX012, 014 and 018 for 24 h. Inset: magnification of cells with vesicles. Scale bar: 50 *µ*m.

We monitored the morphology of HCT116 and SW613-B3 cell lines treated for 24 h with 10 *µ*M BBR, NAX012, 014 and 018 by microscopic observation in bright field. Both cell lines treated with the lead compound BBR did not show relevant alterations in cell morphology, whereas the administration of BBR derivatives was generally accompanied by decreased cell number, rounded morphology, and detachment from the culture substrate (Figure 2). Moreover, the treatment with NAX018 induced the formation of intracellular vesicles, possibly reminiscent of autophagosomes (Inset).

### 3.2. Berberine Derivatives Inhibit Cell Viability

**Figure 3:**
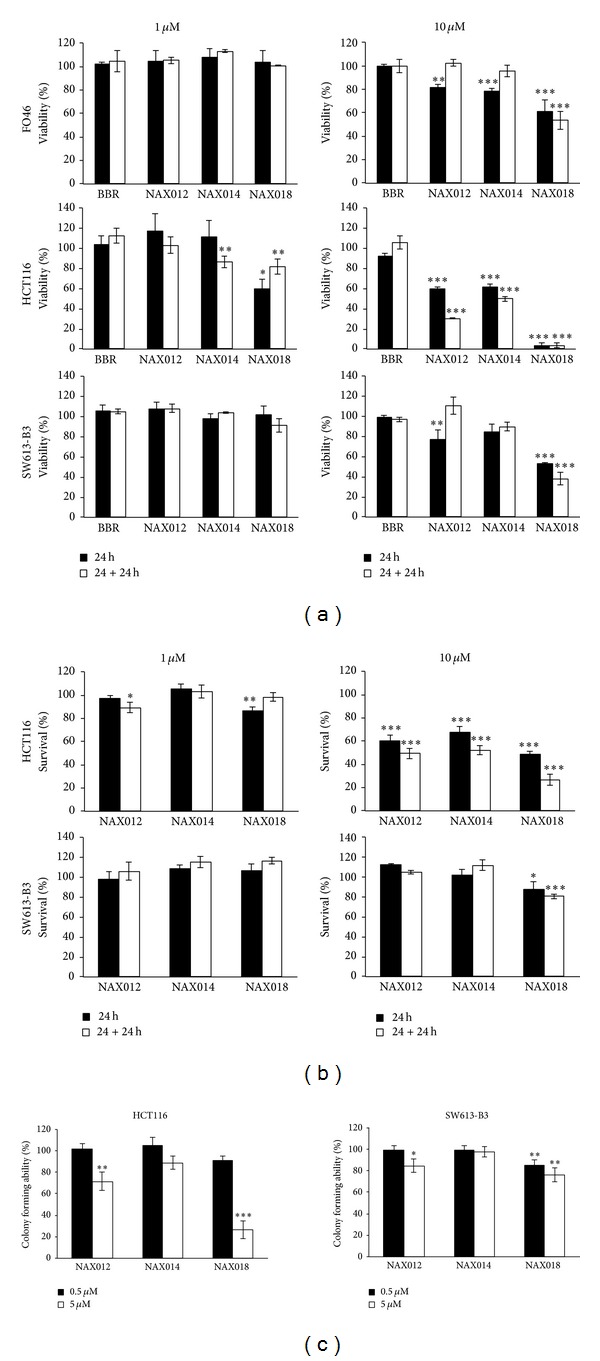
BBR effect on cell survival. (a) MTT metabolic and (b) cytotoxicity assay performed on HCT116 and SW613-B3 cell lines and normal FO46 fibroblasts treated with 1 and 10 *µ*M BBR and NAXs for 24 h (black columns) followed by a 24 h recovery in fresh medium (white columns). (c) Colony forming ability of HCT116 and SW613-B3 cells treated with 0.5 *µ*M (black columns) and 5 *µ*M (white columns) NAXs for 24 h and further grown for 10 days in drug free medium. *P < 0.05; **P < 0.01; and ***P < 0.001.

The MTT assay was applied to tumoral HCT116, SW613-B3 cells, and normal FO46 normal fibroblasts treated for 24 h with 1 and 10 *µ*M BBR, NAX012, 014 and 018 and further grown for 24 h in drug free medium. The proliferation of human normal fibroblasts (FO46) exhibited a modest decrease only after the treatment with 10 *µ*M NAX018 (Figure 3(a)). BBR was ineffective on cancer cells, while a 24 h treatment with NAXs impaired cell viability in a dose-dependent manner and in an irreversible way (Figure 3(a)), with NAX018 being the most effective compound (Figure 3(a)). SW613-B3 cells were more resistant to BBR derivatives than HCT116 cells.

The evaluation of cell survival by a DNA release-based assay revealed that both HCT116 and SW613-B3 cells were not sensitive to 1 *µ*M BBR derivatives (Figure 3(b)). 10 *µ*M NAX012 produced a strong inhibitory effect on HCT116 cell survival (40%) enhanced during the recovery time (50%); NAX014 caused a cell growth inhibition of approximately 30%, which increased to about 50% after the recovery time, while NAX018 showed a cytotoxic effect after the treatment (about 50%) that reached 70% with the recovery time. In SW613-B3 cells, the incubation with 10 *µ*M NAX012 and NAX014 had no inhibitory effect, while NAX018 affected cell survival by 10% and 20% (at the end of the incubation and after the recovery, resp.). Also this assay revealed that HCT116 cells were more susceptible to BBR derivatives.

Accordingly with the previous data, colony forming ability was affected mainly by NAX018 and the effect was more pronounced for HCT116 than for SW613-B3 cells (Figure 3(c)). Altogether, these results point out the dose- and time-dependent cytotoxicity of the three BBR derivatives (in particular NAX018) on HCT116 cells, which are more sensitive than SW613-B3 cells.

### 3.3. BBR Derivatives Impair Cell Cycle Distribution

**Figure 4:**
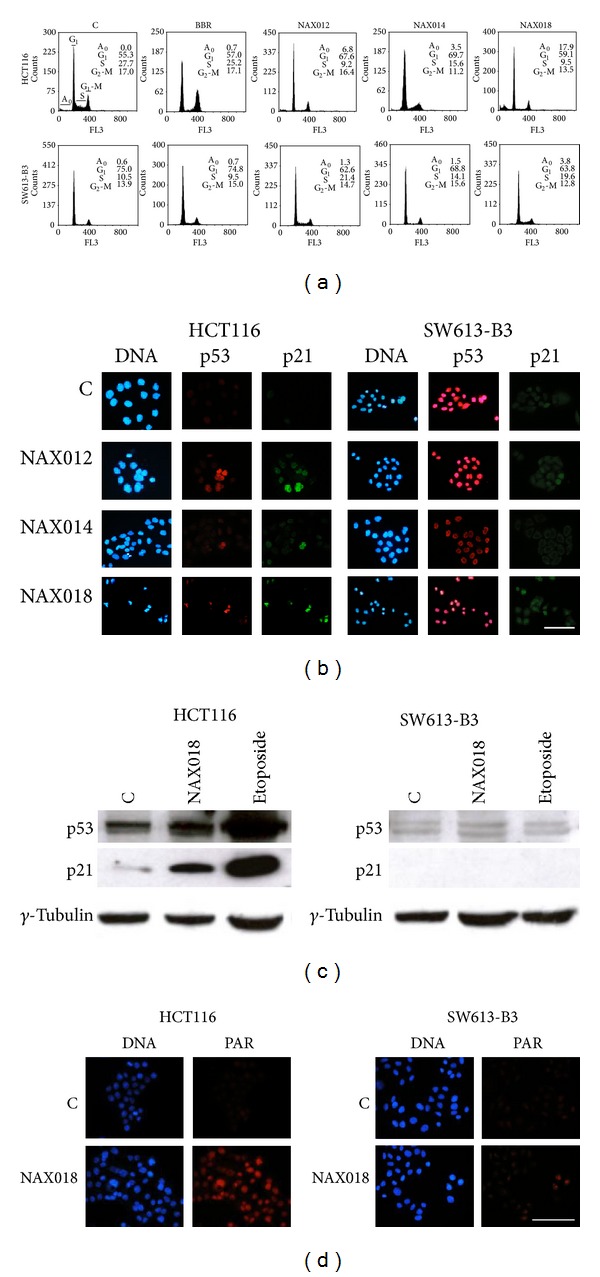
Cell cycle,* p53* and* p21* expression, and PAR accumulation in cells treated with 10 *µ*M NAXs for 24 h. (a) Cell cycle distribution. A_0_: apoptotic cells with DNA content <2 C. (b) Immunolocalization of* p53* (red fluorescence) and* p21* (green fluorescence) in cells treated with NAXs. (c) Western blot analysis of* p53* and* p21* in cells treated with NAX018 and etoposide. (d)* In situ* detection of poly(ADP-ribose) (PAR, red fluorescence). Nuclei were counterstained with Hoechst 33258 (blue fluorescence). Scale bar: 50 *µ*m.

To investigate if the different effect of BBR derivatives on the two cell lines could be ascribed to a diverse impact on cell cycle, we monitored cell cycle distribution by flow cytometry. Cytograms in Figure 4(a) show that BBR was not affecting cell cycle in a significant manner, while HCT116 cells treated with 10 *µ*M NAX012, 014 and 018 tended to arrest cell cycle progression in the G_1_ phase; this phenomenon was accompanied by a decrease in the cell fraction with a DNA content typical of the S phase.

The observed G_1_ arrest could be modulated by* p53*; in fact, we observed an increased immunofluorescent cellular staining of* p53* and* p21* in HCT116 cells treated with BBR derivatives compared to control (C) samples, as expected in a cellular context with functional* p53*, where* p2*1 is necessary for the* p53*-mediated G_1_ arrest (Figure 4(b)). A different behaviour was recorded for SW613-B3 cells, which were not heavily impaired in cell cycle distribution (Figure 4(a)), possibly because of the nonfunctional status of* p53*, which accumulated in untreated cells (C) and was not modulated by the drug treatment, as expected for mutated* p53* in cancer cells [35]; a similar pattern was observed for the protein* p21* (Figure 4(b)). Remarkably, we observed that the labeling of* p53* in SW613-B3 cells was not only confined to the nucleus but was also visible in the extranuclear compartment (Figure 4(b)).

The immunofluorescence data were supported by western blot analysis (Figure 4(c)), revealing that the levels of both* p53* and* p21* proteins increased in drug-treated HCT116 cells but remained very low and unchanged in SW613-B3 cells.

Given that G_1_ arrested HCT116 cells could promote DNA damage, as proved by the data obtained with the comet assay (not shown), we monitored the synthesis of poly(ADP-ribose) (PAR), which is generally formed in response to DNA damage [36].

In fact, by immunofluorescence experiments we observed the accumulation of nuclear PAR in HCT116 cells treated with BBR derivatives (Figure 4(d), red fluorescence). The analysis of the same marker in SW613-B3 cells revealed that PAR synthesis is not stimulated after the treatment with NAX018, possibly because of a low level of DNA damage (Figure 4(d)).

### 3.4. BBR Derivatives Induce Apoptosis

**Figure 5:**
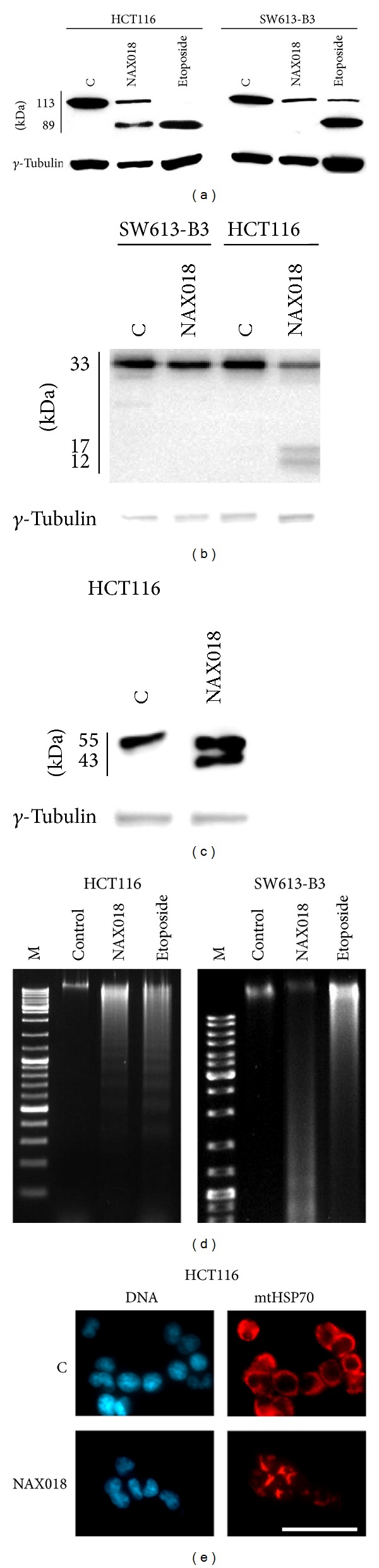
Apoptotic markers. (a) Western blot analysis of PARP-1 cleavage (a), caspase 8 (b) and 3 (c) activation in control cells and in samples treated for 24 h with NAX018 or 100 *µ*M etoposide as a positive control of apoptosis. *γ*-Tubulin was used as protein loading control. (d) Internucleosomal DNA degradation in HCT116 and SW613-B3 cells untreated (control) and treated with NAX018 or 100 *µ*M etoposide; M: DNA molecular marker. (e) Visualization of mitochondria distribution by immunostaining of the mitochondrial HSP70 protein (red fluorescence) in HCT116 cells untreated (C) and treated for 24 h with 10 *µ*M NAX018. Nuclei were counterstained with Hoechst 33258 (blue fluorescence). Scale bar: 50 *µ*m.

Flow cytometry experiments detected a fraction of HCT116 cells with hypodiploid DNA content, thus pointing out the occurrence of apoptosis in cells treated with BBR derivatives. Thus, we analyzed PARP-1 proteolysis, the best apoptotic marker, by western blotting, revealing the expected band at 113 kDa corresponding to the intact protein in both untreated samples (C) and its proteolytic fragment of 89 kDa in drug-treated HCT116 cells. The apoptotic marker was detectable only in SW613-B3 cells treated with etoposide and not with NAX018 (Figure 5(a)), supporting the evidence that proteolytic cascade leading to PARP-1 cleavage was not induced by NAX018 in these cells. Searching for the caspases responsible for PARP-1 cleavage, we observed the presence of the initiator procaspase 8 (33 kDa) in all samples, while the conversion to the active forms (17 kDa and 12 kDa) was detected in response to the treatment of HCT116 cells with NAX018 (Figure 5(b)). Accordingly, the executioner caspase 3 was visible as procaspase (55 kDa) in untreated cells (C) and as the active proteolytic form (43 kDa) in HCT116 cells treated with NAX018. The typical DNA ladder was visualized in HCT116 cells treated with NAX018 and etoposide; DNA degradation occurred also at a lesser extent in SW613-B3 cells (Figure 5(d)).

These biochemical hallmarks were accompanied by a rearrangement in mitochondria distribution, monitored in HCT116 cells by detecting the localization of the mitochondrial HSP70 protein by immunofluorescence experiments. In fact, in untreated cells (C) mitochondria were uniformly distributed throughout the cytoplasm, while in NAX018 treated cells they condensed and formed aggregates (Figure 5(e)).

Altogether, these observations suggest that the cytotoxic effect of BBR derivatives on HCT116 cells is mediated not only by cell cycle arrest but also by the activation of the apoptotic process, possibly mediated by a central role of mitochondria. Conversely, the modest effect recorded for SW613-B3 cells could be ascribed to a low propensity to drive apoptosis.

### 3.5. BBR Derivatives Induce Autophagy

**Figure 6:**
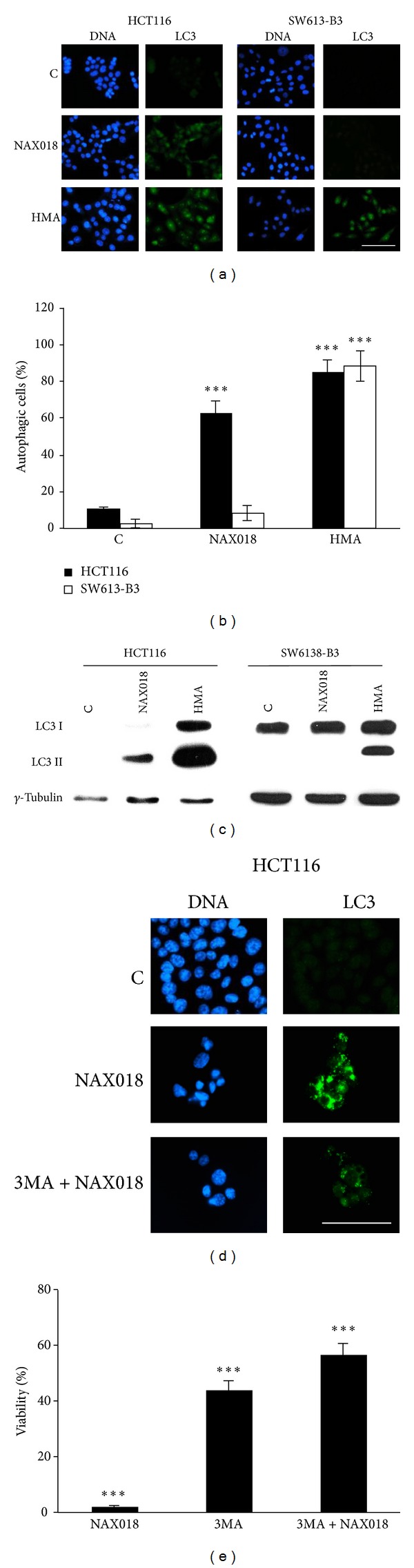
Evidence of autophagy. Immunofluorescence (a) and western blot (C) analysis of the autophagosomal marker LC3 II in HCT116 and SW613-B3 cells untreated (C) and treated for 24 h with 10 *µ*M NAX018. As a positive control of autophagy induction, cells were treated with 20 *µ*M HMA [26]. (b) Quantification of cells with autophagic vacuole punctuation; black columns: HCT116, white columns SW613-B3 cells. Effect of the inhibitor 3MA (2.5 mM for 4 h) on the expression of the autophagic marker LC3 (d) and on cell viability evaluated by the MTT assay in HCT116 cells (e). Scale bar: 50 *µ*m. *γ*-Tubulin was used as protein loading control. ***P < 0.001.

The appearance of vesicles in treated cells (Figure 2) could be suggestive of the possible activation of the autophagic process. To test this hypothesis, we monitored a typical hallmark, that is, the conversion of the protein LC3I into its active form LC3II, by immunofluorescence and western blot. As illustrated in Figure 6(a), a brilliant fluorescent labeling was visible in both cell lines treated with the autophagy inducer HMA [26], while 10 *µ*M NAX018 promoted a very intense staining in HCT116 cells and a faint labeling in SW613-B3 cells. The quantification of cells with autophagic vacuole punctuation is shown in Figure 6(b), where it is evident that SW613-B3 cells are less prone to activate autophagy. This difference was also confirmed by western blot, where the band corresponding to LC3II was intense in NAX018 treated HCT116 cells while undetectable in SW613-B3 cells (Figure 6(c)). The limited ability of SW613-B3 cells to activate autophagy could be correlated to the presence of a pool of* p53* in their cytoplasm (Figure 4(b)). It is well known from the literature that the cytoplasmic localization and translocation of* p53* to mitochondria, possibly mediated by ubiquitylation [37], could influence a number of processes, including autophagy and drug response [38].

To investigate whether cell death induced by BBR could be autophagy-dependent, we pretreated the cells with the autophagy inhibitor 3MA before the administration of the drug. As revealed by MTT experiments (Figure 6(e)), 3MA alone affected cell viability by about 60% and the further addition of NAX018 was no more effective, thus indicating that once the intrinsic autophagy propensity of HCT116 cells is inhibited (as demonstrated by the visualization of LC3 shown in Figure 6(d)), the drug is unable to promote the same amount of cell death as in the absence of 3MA.

In a nutshell, our results demonstrate that the BBR derivatives we synthesized are able to induce cellular DNA damage, in line with an* in vitro* evidence obtained with other 13-substituted BBR [33], and support previous data on their cytotoxicity on breast [34] and colon [39] cancer cells. We describe here for the first time that the BBR derivatives NAX012, 14, and 18 affect cell cycle distribution in a* p53*-dependent manner, in agreement with published data for the lead compound BBR [7, 40–42].

The reported stronger effect of BBR derivatives on HCT116 cells compared to SW613-B3 cells could be attributed to the different status of* p53*, that is, wt and mutated, respectively. The mutation in the codon 273 we described in SW613-B3 cells [25] impairs* p53* ability to bind DNA and to transactivate the main target, that is,* p21* (Figure 4), thus limiting the cytotoxicity of the drug on these cells. This observation is in agreement with the comparison of two prostate cancer cell lines, the one expressing wt* p53* (LNCaP) and the other lacking* p53* (PC3), where BBR was more active on the* p53*-proficient cells; accordingly, the silencing of* p53* in PC3 cells decreased the sensitivity to BBR [18]. The general correlation between the absence of functional* p53* and the low responsiveness to BBR has been reported in neuroblastoma [43], prostate [44], and lung cancer [45] cells.

The most effective compound NAX018 induces apoptosis mainly in HCT116 cells, as supported by caspase 3 activation, internucleosomal DNA degradation, and mitochondria redistribution. These observations are in line with previous reports showing that BBR itself promotes the activation of procaspase 9 and mitochondria deregulation, typically observed in the intrinsic subroutine of apoptosis [19], although in some experimental systems BBR proved to activate the extrinsic pathway [46, 47]. The proapoptotic effect of BBR on cancer cells was reported to be associated with the modulation of JNK/p38-redox/ROS process, HER2/PI3K/AKT signaling,* p53*-regulated factors and NF-*κ*B, AP-1, Wnt, and COX-2 proteins [3, 7, 22, 40, 42].

The evidence of PAR accumulation coupled to mitochondria redistribution prompted us to investigate the occurrence of the caspase-independent cell death paradigm called parthanatos [48] previously reported to be activated by BBR [49]. However, by immunofluorescence experiments we did not detect the translocation of AIF (apoptosis inducing factor) from the mitochondria to the nucleus (not shown), which is the typical hallmark of this type of death.

Finally, we added a piece of information to the recent field of research aiming at investigating the proautophagic power of BBR [22, 50, 51], having recorded for the first time that our BBR derivatives can promote this “Janus” process [52]. The impact of autophagy on the cellular response of cancer cells to BBR derivatives (and on their cytotoxicity) is still under investigation, in order to define if this process could ensure cancer cell survival or act as a form of death, given that two opposite roles have been attributed to it [52].

## 4. Conclusions

The results of the present study indicate that the 13-arylalkyl BBR derivatives NAX012, 014 and 018 have multiple effects on colon cancer cells, extending previous observations on the lead compound BBR [53]. In particular, we found that (i) compared to the lead compound BBR, the NAX compounds are very potent; (ii) they are cytotoxic for two human colon cancer cell lines, being more effective on cells harboring* p53* ^wt^, where they promote cell cycle arrest and DNA damage; and (iii) they trigger caspase-dependent apoptosis and drive autophagy. The above results revealed that the cellular response to NAXs is not univocal, being modulated by* p53*, thus adding further complexity to the pathways governing the effects of BBR (and derivatives) on cancer cells. In fact, most cancers are characterized by a mutated* p53*, which has lost its oncosuppressor function, thus conferring an advantage to cancer cells [35]. Given that we observed that the cell death induced in HCT116 cells by NAX018 is, at least in part, autophagy-dependent, we cannot exclude that the cytoplasmic pool of* p53* visible in the SW613-B3 cell line could be responsible for their drug resistance.

The evidence that the* p53* ^wt^ cancer cell line is susceptible to BBR derivatives is intriguing and legitimates further studies in order to identify the molecular targets of the new NAXs we have developed and characterized. Moreover, our data could help in depicting the molecular pathways governing the beneficial effects of a variety of plant derivatives used in traditional medicine [12, 54–56]. Finally, we have to keep in mind that the global effect of BBR derivatives is strictly dependent on the experimental conditions and cell line tested; to generalize the observation made on colon cancer cells, we aim to extend the analysis to cell lines derived from other tumor types, expressing* p53* either wt or mutated.
